# Association of early and current life factors with telomere length in preterm-born children

**DOI:** 10.1371/journal.pone.0293589

**Published:** 2023-11-08

**Authors:** Ella A. Kotecha, Lei Zhang, Ali Aboklaish, Michael Cousins, Kylie Hart, Sarah J. Kotecha, W. John Watkins, Sailesh Kotecha

**Affiliations:** Department of Child Health, Cardiff University School of Medicine, Cardiff, United Kingdom; Christian Medical College, INDIA

## Abstract

**Background:**

Telomeres shorten after each cell division. Since preterm-born babies are delivered early and often suffer from inflammatory conditions such as bronchopulmonary dysplasia (BPD), their telomere length may be altered.

**Objectives:**

We assessed associations of early and current life factors with telomere length in saliva samples obtained from 7–12-year-old children born at ≤34 weeks’ gestation and term-born controls.

**Study design:**

Relative telomere length was measured by qPCR on extracted DNA. Groups were compared using independent t-tests or ANOVA with post-hoc correction. Linear regression analysis was also used.

**Results:**

534 children had satisfactory telomere data including 383 who were preterm-born (mean (SD) birthweight 1732g (558g), gestation 31.1 (2.6) weeks) and 151 term-born (3464g (510g); 39.8 (1.3) weeks). Telomere length was longer in children who had intrauterine growth restriction (IUGR) at birth: mean (SD): 464.6 (166.3) vs. 418.6 (110.7) in the no-IUGR group; in females: 440.2 (130.1) vs. 405.7 (101.5) in males; and in the least deprived group (397.8 (95.0) vs. 437.6 (121.9) most vs least deprivation quintile). Differences were most notable in females with IUGR. However, telomere length was not different between the preterm and term groups; the BPD and no BPD groups nor was it related to lung function or cardiovascular measurements. In multivariable regression analyses, telomere length was associated with sex, IUGR and deprivation with the greatest difference observed in females with IUGR.

**Conclusions:**

Telomere length was associated with sex, IUGR and deprivation, especially in females with IUGR, but not with prematurity, BPD, lung function or cardiovascular measurements.

## Introduction

Telomeres are the protective, non-coding nucleoprotein structures at the end of chromosomes that consist of TTAGGG repeats connected with Shelterin proteins [1]. In vertebrates, these proteins protect telomeres from DNA repair mechanisms and regulate telomerase activity. They are known to erode with every cell replication to protect the functional, coding DNA from damage, a process known as the ‘end replication problem’ [2] and occurs due to DNA polymerases inability to fully transfer telomeric DNA between strands therefore making their length a potential biomarker of aging [3]. Telomeric erosion is counteracted by telomerase, a ribonucleoprotein reverse transcriptase present in foetal tissue, adult germ, and stem cells, as these enzymes can lengthen chromosomes by adding back the TTAGGG sequences that are lost during cell division [4]. Telomere length has been associated with pathological conditions including atherosclerosis, stroke, myocardial infarction, and type 2 diabetes mellitus [5,6]. Although the associated underlying mechanisms are not clear, inflammation [7] and oxidative stress [8], which are often observed in these pathological conditions, have been shown to result in increased telomere length attrition.

Preterm birth is associated with delivery at an early stage of development of several organs including the lungs, heart, and brain. A recent systematic review reported that preterm birth (including 182 preterm-born and 1,320 term-born newborn infants) was associated with longer telomere length in newborn infants [9] with the length potentially being similar at birth equivalent age [10]. In contrast, intrauterine growth restriction (IUGR) in mainly term-born (227 newborn infants with IUGR and 1,897 without) was associated with shorter telomere length in placental tissue but not in newborn blood possibly reflecting placental aging [9]. Less is known about telomere length in childhood following preterm birth or after IUGR. Whether extrauterine adaptation results in altered cellular replication, thus telomere length, when compared to comparative fetal life is not clear. Furthermore, these babies often suffer from inflammatory conditions including development of bronchopulmonary dysplasia (BPD) [11,12], necrotising enterocolitis, etc. Additionally, how early life factors such as sex, IUGR, social deprivation, and current conditions such as decreased lung function following development of BPD in infancy or cardiovascular abnormalities can alter cellular replication, thus telomere length, is also not clear. We chose early life factors based on previously reported associations between early life factors and telomere length: we included sex, mode of delivery, prematurity as well as those associated with “stressors” including IUGR, BPD and arterial blood pressure. Whilst several of these have been reported previously, there were no clear conclusions regarding these associations most likely due to small numbers in previous studies. We, therefore, hypothesised that the above early and current life factors would result in decreased telomere length in DNA extracted from saliva samples from both preterm- and term-born children with early and current life factors in a large cohort [13].

## Methods

### Population selection & data collection

Children from the Respiratory Health Outcomes in Neonates (RHiNO, EudraCT: 2015-003712-20) study which has previously been described [14] underwent collection of saliva for DNA extraction. Briefly, children from a previous questionnaire study [15,16], (the original questionnaire study invited all survivors of prematurity in Wales who were aged 1,2,3,5,7 and 9 years in 2013 with 7,500 responses), were supplemented with additional preterm-born children, and were mailed a respiratory questionnaire for their parents to complete at 7–12 years of age and if they were geographically accessible and were born at either ≤34 weeks’ gestation or ≥37 weeks’ gestation. Responders to the questionnaire were invited to join RHiNO for a home or hospital visit to obtain anthropometric details, perinatal and respiratory history (supplemented by examination of the child’s medical records) as well as spirometry [13,14]. Children with congenital malformations or significant neurodevelopmental disorders were excluded. A subset of children was invited for more detailed assessments in hopsital including standardised cardiovascular measurements [17,18]. Vicorder (Smart Medical, Gloucestershire, UK) was used to measure central and peripheral arterial blood pressure at rest in a standardised position [18]. Saliva samples were collected from children at the home visit. The children’s parents gave specific consent for DNA collection, and only those who produced an adequate volume of saliva, at the screening or in-depth assessment visit, were included.

BPD was diagnosed according to the National Institute of Child Health and Human Development criteria: if supplemental oxygen was required at 28 days of age, severity was classified at 36 weeks’ post-menstrual age (PMA) for those born at <32 weeks’ gestation or at 56 days of age for those born at ≥32 weeks’ gestation [19]. IUGR was classified as <10^th^ centile for birthweight after adjusting for sex and gestation using LMS growth program [20]. Catchup growth was defined as an increased SD of 0.67 or greater between birthweight and current weight. The spirometry values were quality controlled through the American Thoracic Society/European Respiratory Society guidelines [21], including normalisation against Global Lung Function Initiative reference values [22]. Deprivation values were evaluated using the Welsh Index of Multiple Deprivation (WIMD) scores from 2019 [23], which is a nationally collected, area based deprivation score specific to Wales (similar to the Townsend score of deprivation). The eight area based domains include wealth, education level, home ownership, employment, etc. Individual WIMD quintile score for each child was estimated from their current postcode, with quintile 1 representing the most deprived and 5 the least deprived, and validated against the English Townsend score.

### Saliva sample collection

Saliva samples were collected using the Oragene DNA saliva collection kits (DNA Genotek, Ontario, Canada) [24]. Patients were advised not to eat or drink for 30 minutes prior to collection. 2 mls of saliva was then excreted into a vessel containing a stabilising liquid. The vessel was inverted to ensure through mixing of the stabilising liquid and saliva sample. The samples were stored at -70°C until DNA extraction.

### DNA extraction and qPCR for telomere length

DNA was extracted from the saliva for the preterm group by LGC Genomics (Hertfordshire, UK) as part of wider genome-wide DNA sequencing. DNA extraction from the term-born group was performed using the prepIT-L2P protocol according to the manufacturer’s instructions (DNA Genotek, Ontario, Canada). Relative telomere length per diploid cell was quantified against telomere length standards using a commercially available kit qPCR assay according to the manufacturer’s instructions (AHTLQ, ScienCell, California, USA). The mean intra- and inter-observer coefficients of variation within and between qPCR plates were 1.5% and 2.0% respectively.

### Statistical analysis

We had aimed to collect 500 DNA samples including 350 from preterm born children and 150 term born children. Two-tailed independent t-tests and one-way ANOVA tests with post-hoc Bonferroni correction were used to compare groups. Univariable and multivariable linear regression was used to determine associations between early/current life factors and telomere length. p<0.05 was considered statistically significant. All data analysis was performed using IBM SPSS statistics version 27 (IBM, NY, USA).

## Results

Participant demographics are shown in Table 1. From 534 children with satisfactory telomere data, 383 were born preterm mean (SD): birthweight 1732g (558g); gestation 31.1 (2.6) weeks and 151 were term-born: birthweight 3464g (510g); gestation 39.8 (1.3) weeks. 68 (18.0%) of the preterm group had BPD (including 27 (39.7%) with mild and 41 (60.3%) with moderate/severe BPD); and 53 (10.0%) had IUGR including 46 (12.0%) in the preterm group and 7 (4.6%) in the term group. As expected, the preterm group had significantly lower birthweight and gestation; and higher rates of IUGR and BPD. Height, weight, and BMI were similar at time of telomere length assessment including between boys and girls. Seven children did not have satisfactory spirometry data when quality controlled against ATS/ERS guidelines [25]. When the preterm and term groups were compared, percent predicted forced expiratory volume in 1 second (FEV_1_) (mean (SD) 90.8% (13.4%) and 95.4% (9.8%), p = 0.002); percent predicted forced expiratory flow at 25–75% (FEF_25-75%_) (76.5% (21.5%) and 85.6% (19.7%) respectively, p<0.001) and for FEV_1_/FVC ratio (0.84 (0.08) and 0.86 (0.07) p = 0.018) were decreased but percent predicted forced vital capacity (FVC) (94.2% (11.5%) and 96.4% (10.0%) p>0.05) was similar. 202 (141 preterm- and 61 term-born) children underwent standardised blood pressure measurements, with only one child not obtaining satisfactory central blood or pulse pressure measurments. The central (mean (SD): 112.7 (9.6) and 108.3 (10.1) mmHg, p = 0.003 for the preterm and term groups respectively) and peripheral systolic (mean (SD): 120.8 (9.8) and 117.2 (11.2) mmHg, p = 0.021) blood pressure were significantly increased, and the central (58.1 (7.6), 56.0 (6.6) mmHg, p = 0.074) and peripheral (58.1 (7.6) and 56.0 (6.6) mmHg, p = 0.067) diastolic blood pressure were non-significantly increased, in the preterm group when compared to the term group, respectively.

**Table 1:** Participant demographics for the preterm and term groups.

			Preterm Group (N = 383)	Term Group (N = 151)
**Early Life Factors**				
Male N (%)			190 (49.6%)	74 (49.0%)
Birthweight (gram)(SD)			1732 (558)	3464 (510) ¶¶¶
Gestational Age (weeks)(SD)			31.1 (2.6)	39.8 (1.3) ¶¶¶
IUGR N (%)			46 (12.0%)	7 (4.6%) ¶
BPD N (% of preterm population)			68/378 (18.0%)	0 (0%)¶¶¶
**Mode Of Delivery**	Spontanous Vaginal Delivery (%)		171 (44.6%)	116 (76.8%)
	Lower Segment Caesarean Section (%)		210 (54.8%)	35 (23.2%)
Antenatal Smoking N (%)			49/377 (13.0%)	9/151 (6.0%)¶
**Current Factors**				
Age (SD)			10.1 (1.1)	9.8 (1.1)
Current Weight (kg)(SD)			37.6 (10.9)	37.4 (10.0)
Current BMI (SD)			18.2 (3.5)	18.2 (3.2)
Current Height (cm)(SD)			142.5 (9.8)	142.6 (9.4)
WIMD 2019 N (%)	1—Most Deprived	Proportion Mean WIMD score	61/383 (15.9%) 188.1 (115.3)	12/151 (7.9%) 166.8 (109.0)
	2	Proportion Mean WIMD score	70/383 (18.3%) 575.5 (108.3)	22/151 (14.6%) 569.2 (113.8)
	3	Proportion Mean WIMD score	70/383 (18.3%) 964.3 (119.6)	32/151 (21.2%) 973.4 (119.4)
	4	Proportion Mean WIMD score	76/383 (19.8%) 1357.5 (113.0)	30/151 (19.9%) 1316.3 (100.3)
	5 -Least Deprived	Proportion Mean WIMD score	106/383 (27.7%) 1751.5 (104.0)	55/151 (36.4%) 1742.6 (115.0)
		
**Lung Function**				
N			378	149
Percent Predicted FEV_1_ (SD)			90.8% (13.4%)	95.4% (9.8%) ¶¶
Percent Predicted FVC (SD)			94.2% (11.5%)	96.4% (10.0%)
Percent Predicted FEF_25-75%_ (SD)			76.5% (21.5%)	85.6% (19.7%) ¶¶¶
FEV_1_/FVC Ratio			0.84 (0.08)	0.86 (0.07) ¶¶
		
**Cardiovascular Measurements**				
N			141	61
Central Systolic Blood Pressure mmHg (SD)			112.7 (9.6)	108.3 (10.1) ¶¶
Central Diastolic Blood Pressure mmHg (SD)			58.1 (7.6)	56.0 (6.6)
Peripheral Systolic Blood Pressure mmHg (SD)			120.8 (9.8)	117.2 (11.2) ¶
Peripheral Diastolic Blood Pressure mmHg (SD)			58.1 (7.6)	56.0 (6.6)
Central Pulse Pressure mmHg (SD)			54.7 (10.1)	52.3 (9.3)

Abbreviations: BMI—body mass index, IUGR—intrauterine growth restriction, BPD—bronchopulmonary dysplasia, FEV_1_ –forced expiratory volume in 1 second, FVC–forced vital capacity, FEF25-75%–forced expiratory flow at 25–75%, WIMD–Welsh index of multiple deprivation.

^¶^ denotes p<0.05,

^¶¶^ p<0.01 and

^¶¶¶^ p<0.001 when the preterm and term groups were compared.

**Fig 1:**
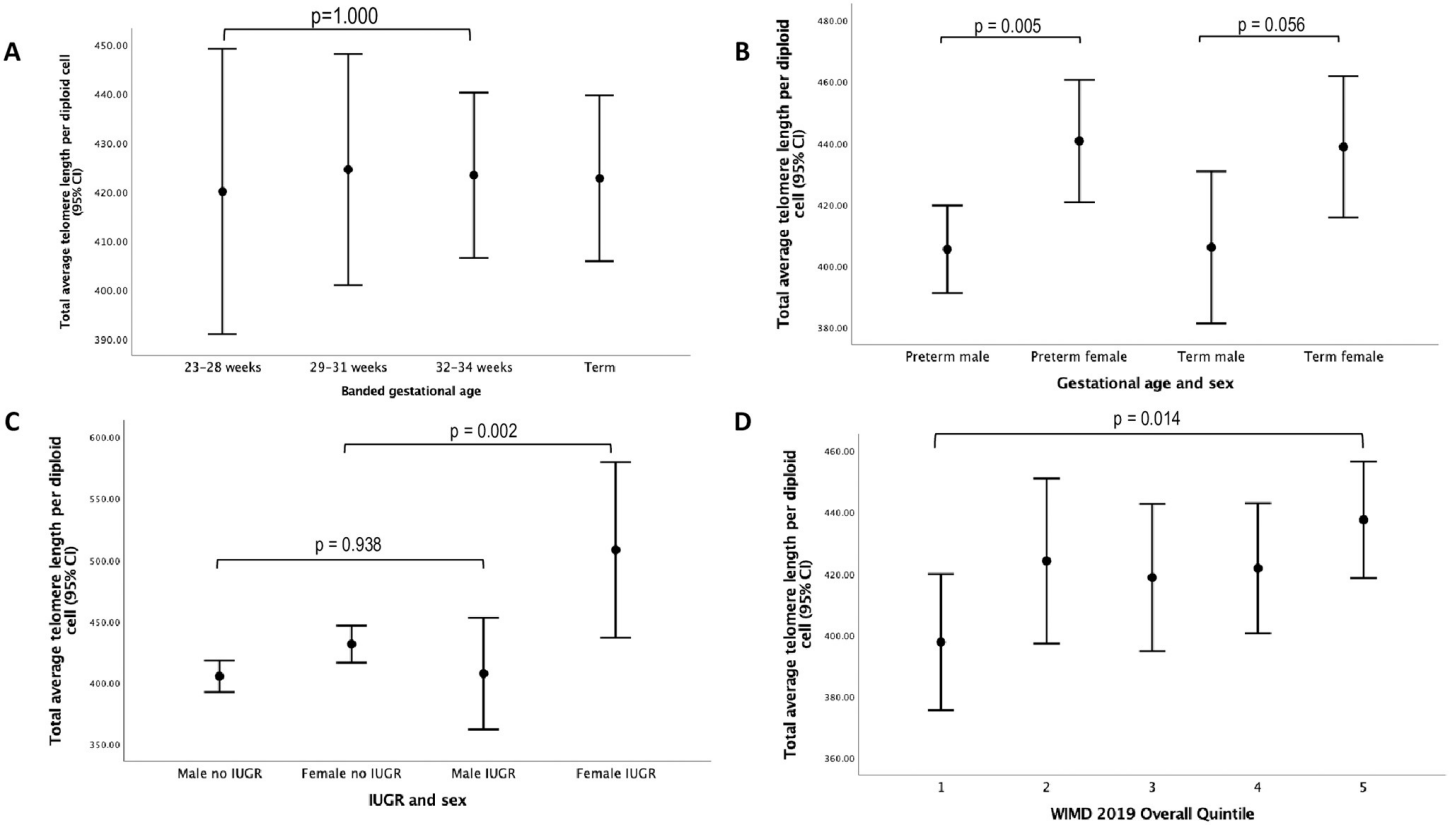
Relationship between telomere length in each diploid cell and (a) banded gestation; (b) sex and prematurity; (c) sex and IUGR; and (d) deprivation measured by the Welsh index of multiple deparivation (WIMD).

The mean (SD) telomere length data are shown In Table 2. There were no differences in telomere length between the preterm and term groups (423.3 (122.8) and 422.8 (105.0), p = 0.968 respectively). In addition there were no differences noted when we banded the gestations (23–28, 29–31, 32–34 weeks’ gestation and term group) Fig 1A. Mode of delivery did not show a difference between the groups in whole group nor when the term and preterm groups were analysed separately: spontaneous vaginal delivery: 420.6 (107.9) and lower segment caesarean section: 462.2 (129.3), p = 0.586 for whole popualtion.

**Table 2:** Mean telomere length per diploid cell and the standard deviations associated with early and current life factors. Comparisons were made BPD groups and gestation bands with telomere length using independent sample t-tests and one-way ANOVA with Bonferroni post hoc correction.

		Whole group (N = 534)	P values	Preterm Group (N = 383)	p values	Term Group (N = 151)	p values
Whole Group		423.1 (118.0)		423.3 (122.8)	P = 1.000	422.8 (105.0)	P = 0.968
23–28 weeks (n = 60, 11.2%)				420.1 (112.5)			
29–31 weeks (n = 122, 22.8%)				424.6 (131.4)			
32–34 weeks (n = 201, 37.5%)				423.4 (121.0)			
Sex	Male (n = 264)	405.7 (101.5)	<0.001	405.5 (99.6)	0.005	406.1 (106.9)	0.056
	Female (n = 270)	440.2 (130.1)		440.7 (140.1)		438.8 (101.3)	
BPD	Mild BPD (n = 27)			380.2 (82.7)	0.287		
	Moderate/Severe (n = 41)			428.2 (109.7)			
	No BPD (n = 310)			425.6 (126.4)			
IUGR	IUGR (n = 53)	464.6 (166.3)	0.007	473.8 (175.1)	0.003	404.7 (69.2)	0.642
	No IUGR (n = 481)	418.6 (110.7)		416.4 (112.5)		423.7 (106.5)	
WIMD 2019	Q1 (most deprived)	397.8 (95.0)	0.014	395.2 (99.2)	0.010	410.8 (72.4)	0.676
	Q5 (least deprived)	437.6 (121.9)		443.7 (123.7)		425.8 (118.6)	

All telomere lengths per diploid cell are represented as mean (SD). Abbreviations: BPD = Bronchopulmonary Dysplasia; IUGR = intrauterine growth restriction; WIMD = Welsh multiple index of deprivation.

**Fig 2:**
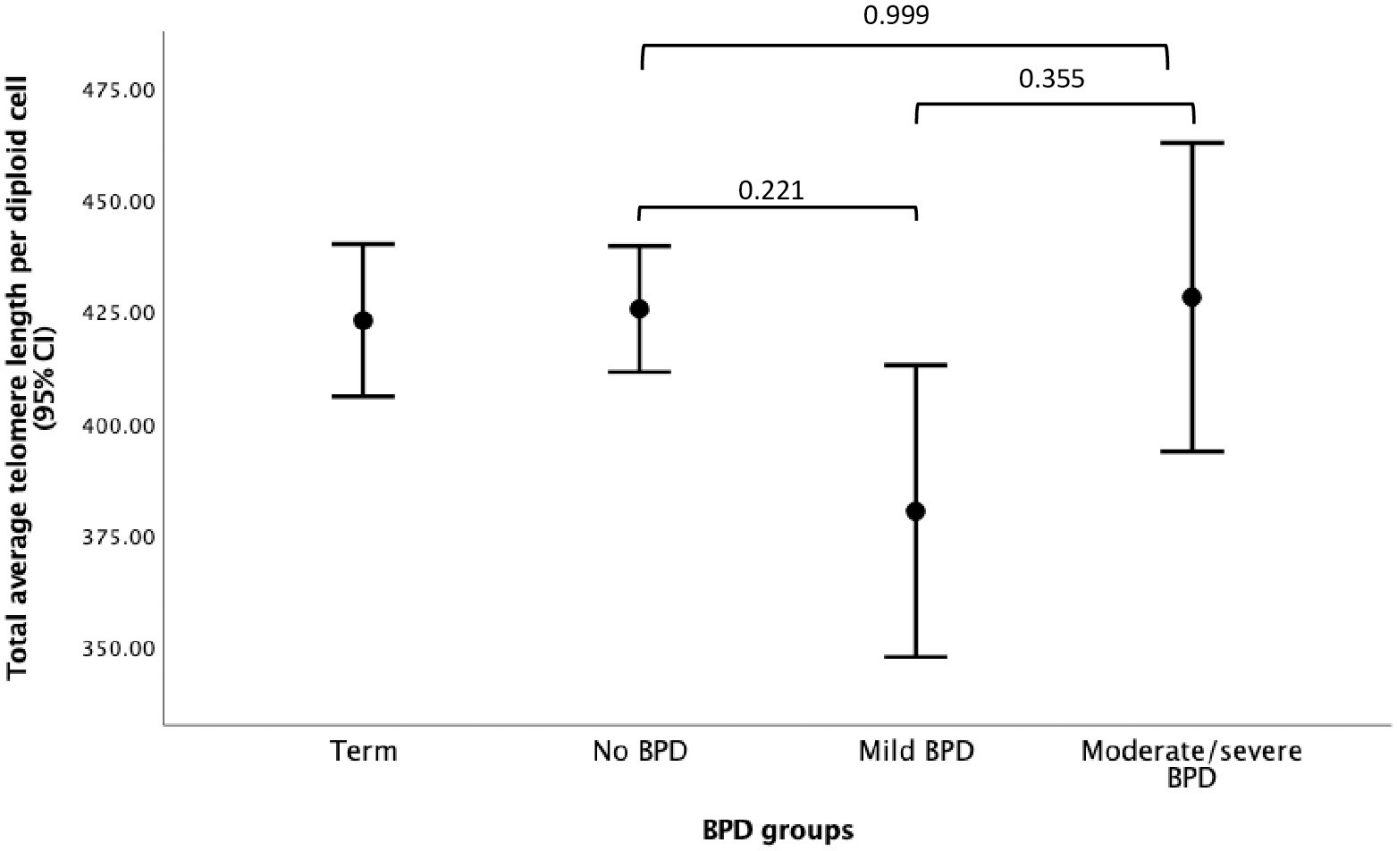
Relationship between telomere length in each diploid cell and BPD severity.

However, females had longer telomere length than males (440.2 (130.1) and 405.7 (101.5) respectively, p<0.001) and those born with IUGR also had longer telomere length (464.6 (166.3) and 418.6 (110.7) respectively, p<0.007). On further investigation, the telomere lengths were greater in females than in males in both the preterm and term groups (Fig 1B), but IUGR was associated with greater telomere length in females but not in males with IUGR when compared to either sex without IUGR (Fig 1C). Using ANOVA with post-hoc corrections for the whole population, a significant difference was noted between the no IUGR (418.6 (110.7), n = 481) and IUGR catch-up group (462.9 (177.9), p = 0.044, n = 44). However, although the telomere length was greater in the IUGR no catch up group (473.2 (96.9)) when compared to the no IUGR group (418.6 (110.7)), this difference was not significant (p = 0.350) possibly due to only 9 subjects available for inclusion in this group. Interestingly, from the whole population those who were from the most deprived group had shorter telomere length than those from the least deprived group (397.8 (95.0) and 437.6 (121.9) respectively, p = 0.014) (Fig 1D). There was no difference between the mild or moderate/severe BPD groups when compared with the preterm or term groups (Table 2 and Fig 2).

We next examined if there was a relationship between spirometry measures including %FEV_1_, %FEF_25-75%_, %FVC and FEV_1_/FVC and cardiovascular measurements including central and peripheral systolic and diastolic blood pressure measures as well as prematurity, sex, IUGR, BPD and deprivation with telomere length using univariable linear regression (Table 3). Spirometry and blood pressure measures, as well as prematurity and BPD, were not associated with telomere length but sex, IUGR and deprivation were. On multivariable regression analyses, all three included variables of sex, IUGR and deprivation remained significantly associated with telomere length (Table 3). Finally, we showed interactions between sex and IUGR in both the total cohort and in the preterm group, which showed that, compared to females without IUGR, females with IUGR had longer telomere length but males without IUGR had significantly shorter telomere length; and males with IUGR had non-significantly deceased telomere length (Table 3).

**Table 3:** Displays the univariate and multivariate analysis of the variables.

**Univariate Analysis Of Potential Predictors Of Decreased Telomere Length**					
		b	SE / 95% CI	p-value	N (%)
**Factors**					
IUGR (reference = no IUGR)		46.1	(12.9, 79.3)	0.007*	534
sex (reference = male)		34.5	(14.7, 54.3)	<0.001*	534
Prematurity (reference = term)		0.46	(-21.7, 22.7)	0.967	534
BPD	BPD	-13.9	(-47.6, 19.9)	0.421	527
	No BPD	2.5	(-20.4, 25,5)	0.829	
	Term	Reference group			
					
**WIMD**	1 (most deprived)	Reference group			534
	2	26.4	(-9.6, 62.4)	0.151	
	3	21.0	(-14.2, 56.2)	0.242	
	4	24.0	(-10.9, 58.9)	0.178	
	5 (least deprived)	39.8	(7.4, 72.2)	0.016	
**COVARIATES**					
				
**Lung Function**					
%FEV_1_		-0.036	0.41	0.930	527^1^
%FEF_25-75%_		-0.10	0.24	0.678	527^1^
%FVC		0.11	0.46	0.820	527^1^
Fev_1_/fvc ratio		-16.4	67.8	0.809	527^1^
				
**Cardiovascular Measurements**					
Central Systolic BP		-0.38	0.95	0.689	201^2^
Central Diastolic BP		0.63	1.28	0.625	201^2^
Peripheral Systolic BP		-0.09	0.91	0.919	202
Peripheral Diastolic BP		0.46	1.28	0.721	202

**Multivariable Modelling Of The Significant Univariate Variables**					
		b	95% CI	p-value	N
**FACTORS**					
Sex (reference = male)		33.0	(13.4, 52.6)	<0.001*	534
IUGR (reference = no IUGR)		43.4	(10.5, 76.4)	0.010*	534
WIMD	1 (most deprived)	Reference			534
	2	25.2	(-10.2, 60.6)	0.163	
	3	17.1	(-17.6, 51.8)	0.335	
	4	22.2	(-12.2, 56.6)	0.206	
	5 (least deprived)	37.3	(5.4, 69.2)	0.022	

**Interactions Between IUGR And Sex (Total Cohort)**					
		b	95% CI	p-value	
Female*No IUGR		Reference			
Female*IUGR		76.6	(-32.9, 120.4)	<0.001*	
Male*No IUGR		-26.2	(-46.8, -5.6)	0.013*	
Male*IUGR		-24.0	(-73.3, 25.3)	0.341	
			
**Interactions Between IUGR And Sex (Preterm Group Only)**					
		b	95% CI	p-value	
Female* IUGR		Reference			
Female* No IUGR		-92.8	(-141.3, -44.3)	<0.001*	
Male* IUGR		-113.2	(-183.2, -43.3)	0.002*	
Male* No IUGR		-115.2	(-163.6, -66.8)	<0.001*	

All covariates are presented as b and SE, all factors are presented as b and 95% CI. An Asterix (*) represents a significant result, where p < 0.05.

For the interactions between IUGR and sex an Asterix (*) represents a significant result, where p < 0.05.

Abbreviations: IUGR = intrauterine growth restriction, BPD = Bronchopulmonary Dysplasia, WIMD = Welsh multiple index of deprivation, BP = blood pressure, %FEV_1_ = percent predicted forced expiratory volume in 1 second, %FVC = percent predicted forced vital capacity, %FEF_25-75%_ = percent predicted forced expiratory flow at 25–75%.

^1^Seven children did not satisfy ATS/ERS spirometry criteria.

^2^Central blood pressure was not available for one child.

## Discussion

In this study, we noted that telomere length in diploid cells was increased in females in childhood when compared to males although the difference for the term group just failed to reach statistical significance (p = 0.056); increased in those with IUGR when compared to those without IUGR; and was increased in those in the least socioeconomically deprived group when compared to the most deprived group. In contrast, telomere length was similar in the preterm group when compared to the term group. Despite differences in spirometry and blood pressure measurements between the preterm and term groups, no differences were noted for telomere length using univariable regression analyses. When the differences were investigated further, the increased telomere length seemed to be confined to females with IUGR in the preterm group but not in males with IUGR when compared to the respective no IUGR groups. Furthermore, telomere length was increased in females regardless of being preterm or term.

It was clear that females had increased telomere length in childhood than males regardless of whether they were born preterm or term, although just failed to reach significance in the term group. Previous studies have produced conflicting results. Some have reported longer telomere length in females than males at birth [26–30], but others have reported no significant differences between female and males [10,31,32]. The increased length has been speculated to be due to increased levels of oestrogen, which may provide a protective effect on telomeres. Females also have greater telomerase activity than males (26) which may increase the rate at which telomeres can be replenished.

The results for association between telomere length at birth and IUGR are conflicting as recently reported in a systematic review [9]. The systematic review concluded that telomere length was shortened at birth in those with IUGR when compared with those without IUGR but only when telomere length was measured in placental samples but not when measured in neonatal blood. However, our study was conducted in school-aged children. We noted increased telomere length in females with IUGR when compared to females without IUGR but no difference was noted for males with and without IUGR. These differences were noted despite the somatic growth in childhood being similar between the groups, including between boys and girls. Whether the increased telomere length reflects potential for additional cell replication is speculative and will need longitudinal assessment to determine if the increased length persists into adulthood. Interestingly, in our analysis the statistical difference between sex and IUGR was much more significant within the preterm group than in the whole cohort. This may be due to an increased potential for catch up growth in the preterm group. Upon further analysis, no significant difference was noted between the no IUGR (418.6 (110.7)) and either the catch up (462.2 (117.9), p = 0.044) or no catch up (473.2 (96.9), p = 0.350) IUGR groups. Although our catch up group was small (n = 9), further research into this area may reveal more conclusive results.

There was no difference for telomere length in the preterm or term groups. In the newborn, clear differences have been noted between the preterm and term groups but there is some evidence to show that telomere length shortens by term corrected age [10]. Interestingly, the pulmonary inflammatory disease, BPD in infancy, was not associated with differences between the BPD (including in the moderate/severe BPD group) and no BPD or term groups despite clear spirometry deficits in the preterm group when compared to the term group or between the BPD and preterm groups as we have previously reported (13). These findings contrast with previous studies which have reported associations between small airway disease and telomere length. Hadchouel *et al* reported a strong correlation between forced expiratory flow 25–75% (FEF_25-75%_) in extremely preterm born adolescents, this was attributed to continuing airway oxidative stress (27). Henckel *et al* also reported a decrease in FEF_25-75%_ with decreased relative telomere length, however this was only significant in the term born group who had asthma [33]. This difference was attributed to the potential systemic eosinophilic inflammation. Telomere length attrition has been shown to be associated with cardiovascular disease in adulthood [34]. However, despite noting increased central and peripheral systolic pressure, we did not note any associations between central and peripheral systolic blood pressure and other measures with telomere length.

We also observed shortened telomere length in the most socioeconomically deprived population when compared to the least deprived population. There is paucity of data on the association between telemore length and socioeconomic status in preterm born children. Previous studies have shown that low socioeconomic status contributes towards accelerated cell growth during childhood [35]. The underlying reasons for the the shortened telomere length in our population are unclear but one can speculate that this may be due to factors associated with deprivation, such as exposure to smoking and nutrition, although obesity is also associated with deprivation. In addition, longitudinal studies will be required to determine if this is aprogressive or static state in this group of children.

In this study, we used less invasive saliva samples over other invasive methods, e.g., blood samples due to their acceptability and likelihood of greater success. Previous studies have shown that saliva’s composition is approximately 80% lymphocytes and that telomere length measured in DNA extracted from saliva correlates well with peripheral blood samples (R = 0.96, p<0.001) [36]. qPCR was chosen as the method of telomere length measurement over the other common methods of telomere restriction fragment (TRF) or flow-FISH. TRF indirectly measures telomere length using a modified Southern blot technique to measure the range of telomere lengths within a cell and can over-estimate telomere length. Flow-FISH is a combination of fluorescent *in situ* hybridization and flow cytometry, the measure of fluorescence is correlated with the accurate median telomere length of the cell population. The qPCR method was chosen as the benefits outweighed the drawbacks, in comparison to the other methods [37]. We also studied the largest population thus far allowing more robust study of associations with early and current life factors.

In summary, we show that there were no differences for telomere length between preterm and term born children but noted that telomere length was increased in females especially in those who had IUGR at birth. Despite differences in spirometry and for blood pressure measures, the telomere length was similar in childhood, but deprivation was associated with shortened length in the most deprived group when compared to the least deprived group.
